# Validating Combination Throat-Nasal Swab Specimens for COVID-19 Tests Would Improve Early Detection, Especially for the Most Vulnerable

**DOI:** 10.1093/cid/ciae381

**Published:** 2024-07-23

**Authors:** Alexander Viloria Winnett, Timothy Stenzel, Rustem F Ismagilov

**Affiliations:** Division of Chemistry and Chemical Engineering, California Institute of Technology, Pasadena, California, USA; Division of Biology & Biological Engineering, California Institute of Technology, Pasadena, California, USA; California Institute of Technology Medical Scientist Training Program, University of California Los Angeles, Los Angeles, California, USA; Office of In Vitro Diagnostics, US Food and Drug Administration, Silver Spring, Maryland, USA; Division of Chemistry and Chemical Engineering, California Institute of Technology, Pasadena, California, USA; Division of Biology & Biological Engineering, California Institute of Technology, Pasadena, California, USA

**Keywords:** COVID-19, validation testing, early detection, rapid diagnostics, specimen types

## Abstract

Early detection of severe acute respiratory syndrome coronavirus 2 infection by diagnostic tests can prompt actions to reduce transmission and improve treatment efficacy, especially for vulnerable groups such as immunocompromised individuals. Recent evidence suggests that sampling the throat in addition to the nose improves clinical sensitivity during early infection for both antigen and molecular coronavirus disease 2019 tests. We urge test manufacturers to validate tests for use with throat swab, in combination with nasal swabs.

Individuals with immunocompromise and other vulnerable groups at high risk for severe disease continue to rely heavily on coronavirus disease 2019 (COVID-19) testing. This reliance often includes screening contacts before in-person interactions, to prevent the risk of exposure to individuals with presymptomatic or asymptomatic infections. Even in the absence of symptoms or known exposure, individuals with immunocompromise may also test themselves regularly to identify early infection and quickly initiate treatment. For this population—approximately 7 million people in the United States with primary immunodeficiencies or immunosuppressive treatment for cancer, transplants, or autoimmune disorders [1]—tests that detect early infection with high sensitivity are essential.

Among COVID-19 tests, the low cost, direct-to-consumer sale, and rapid results of at-home antigen rapid diagnostic tests (Ag-RDTs) make them an attractive and increasingly used diagnostic modality both for high-risk individuals and the general population [2, 3]. While the US Food and Drug Administration (FDA) has long been open to throat swab specimens for COVID-19 testing, all at-home Ag-RDTs are currently authorized only for use with self-collected nasal swab specimens [4]. However, nasal swab specimen Ag-RDTs have been demonstrated to have low to moderate (approximately 50%–80%) clinical sensitivity to detect infection in individuals, especially those who are asymptomatic and/or in the early stage of infection [5], when transmission of severe acute respiratory syndrome coronavirus 2 (SARS-CoV-2) often occurs [6].

Several cross-sectional studies have demonstrated that Ag-RDTs exhibit higher clinical sensitivity when a combination of nasal (anterior nares) and throat (posterior oropharynx plus palatine tonsils) swabbing is used, compared with a nasal swab specimen alone (Figure 1*A*). A small study in Nova Scotia evaluated the use of combined nasal and throat swabbing for 2 separate Ag-RDTs (Panbio COVID-19 Ag Rapid Test Device and BTNX Rapid Response COVID-19 Antigen Rapid Test) in asymptomatic individuals [7]. Among 62 and 40 infected individuals respectively, 24% (Panbio) and 18% (BTNX) improvements in clinical sensitivity were observed by combining nasal and throat Ag-RDT results, compared with nasal-specimen-only Ag-RDT results. This study also demonstrated a 13% increase in clinical sensitivity by testing a single combined throat-nasal swab specimen compared with nasal swab specimen alone in 38 infected individuals. A separate, large study of 827 infected individuals in Copenhagen, Denmark, recently demonstrated that combined nasal and throat Ag-RDT results improved clinical sensitivity by upward of 16% compared with nasal Ag-RDT results alone [8].

**Figure 1.**
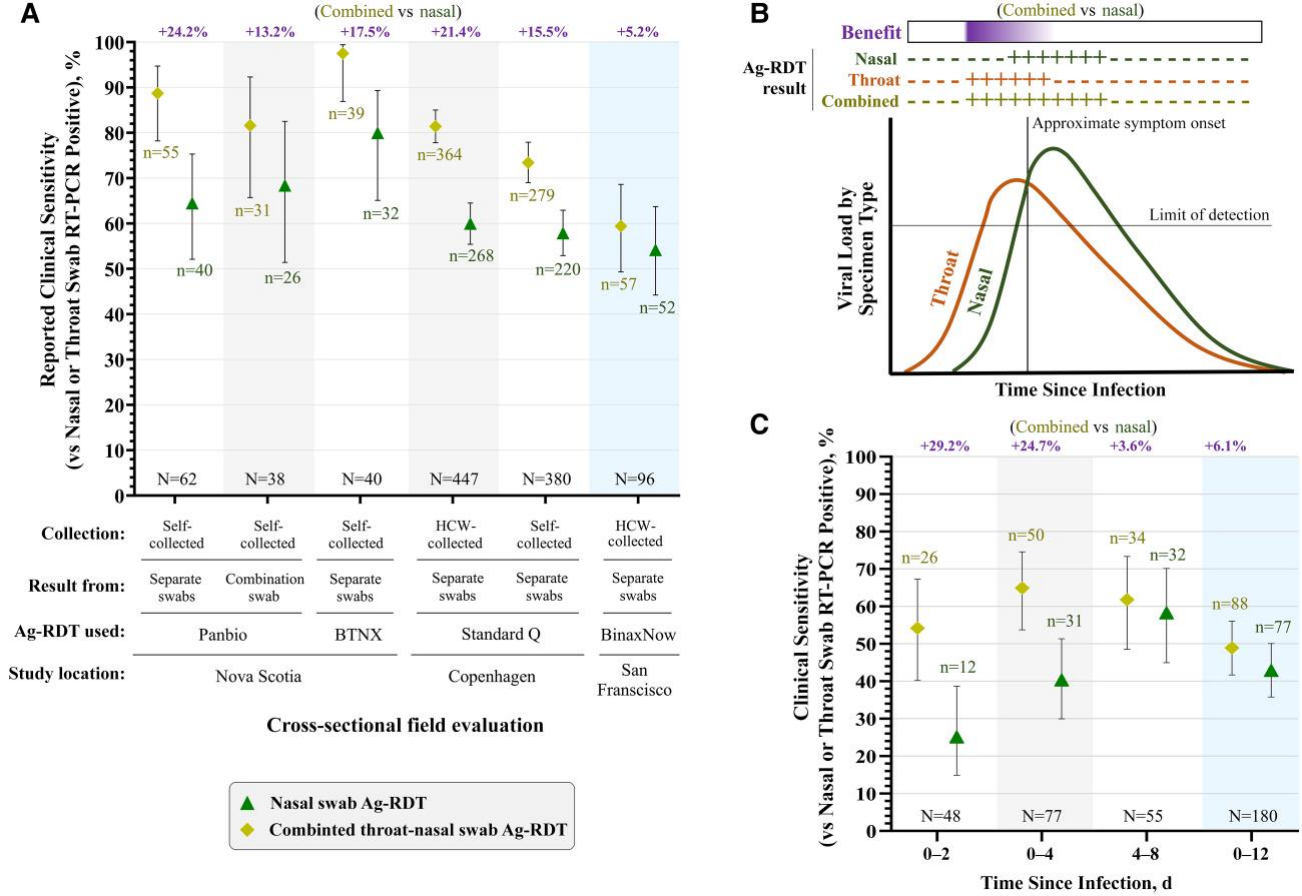
*A*, Summary of studies reporting the clinical sensitivity of combined throat-nasal swab specimen antigen rapid diagnostic tests (Ag-RDTs) compared with nasal-specimen-only Ag-RDTs. The difference between the clinical sensitivity of combined throat-nasal swab specimen Ag-RDT results and nasal-specimen-only Ag-RDT results is shown in purple above each plot. Data are reproduced from cross-sectional field evaluations in Nova Scotia, Canada [7], Copenhagen, Denmark [8], and San Francisco, California [9]. These field evaluations had slight differences in design. “HCW collected” indicates specimen collection by a healthcare worker; “self-collected,” collection by the study participant. “Separate swabs” refers to designs in which test results represent the composite outcome of testing nasal and throat swabs separately; “combination swab,” designs in which the result was determined by directly testing a single swab used to sample both the nose and throat. Abbreviations: RT-PCR, reserve-transcription polymerase chain reaction. *B*, Conceptual schematic depicting the typical presentation of longitudinal severe acute respiratory syndrome coronavirus 2 (SARS-CoV-2) viral loads in nasal and throat swab specimens from the beginning of infection (first positive high-analytical-sensitivity test result in any specimen type), based on data from a study of individuals with naturally acquired infection in Los Angeles, California [10] and individuals inoculated with SARS-CoV-2 in London, United Kingdom [11]. The hypothetical nasal, throat, and combined throat-nasal Ag-RDT results are expected based on this typical presentation of viral loads, to illustrate why the increased clinical sensitivity of combined throat-nasal Ag-RDT over nasal-specimen-only Ag-RDT would be greatest during early in infection and wane during later infection. Horizontal line indicates the limit of detection for Ag-RDTs. *C*, Clinical sensitivity of combined throat-nasal Ag-RDT (inferred from viral loads) and nasal-specimen-only Ag-RDT results (participant reported) during different periods of infection, based on data from field evaluation of nasal swab specimen Ag-RDT with paired viral load quantification in Los Angeles [5]. Rightmost conditions (blue shading) in *A* and *C* highlight how cross-sectional evaluations that include time points late in infection may underestimate the benefit of combined throat-nasal Ag-RDT over nasal-specimen-only Ag-RDT.

Longitudinal viral load data suggest that infection stage influences the magnitude of the benefit of combined throat-nasal specimen Ag-RDT compared with nasal-specimen–only Ag-RDT. Daily viral loads quantified from prospectively collected nasal and throat swab specimens in individuals with incident SARS-CoV-2 infection revealed that the virus often presents in the throat days before presenting in the nose [10]. A simplified representation based on available data [10, 11] for the typical presentation of viral loads in the throat and the nose during early infection illustrates how the benefit of adding throat swab specimens to nasal specimen Ag-RDTs is expected to be greatest during the first few days of infection (Figure 1*B*). Indeed, based on quantitative viral load measurements in the throat and nose during the first 4 days of infection, Viloria Winnett et al [5] predicted that a combined throat-nasal specimen Ag-RDT would have approximately 25% greater clinical sensitivity than a nasal-specimen-only Ag-RDT (Figure 1*C*). This prediction was similar to the benefits observed in the later studies performed in Nova Scotia, Canada [7], and Copenhagen [8]. In addition, supplemental data from Copenhagen shows that the benefit of combined throat-nasal Ag-RDT results over nasal-specimen-only results decreased with time from symptom onset among individuals from whom healthcare workers collected specimens, from 32% on the first day of symptoms to 13% thereafter [8].

The benefit of combined throat-nasal sampling extends to molecular COVID-19 tests as well. Among 14 individuals with naturally acquired incident SARS-CoV-2 infection, 10 (71%) had viral loads >1000 copies/mL in throat swab specimens for at least a day before viral loads in the nose rose to this level [10]. For many individuals, the delay was longer; more than a third of participants (5 of 14) had virus in the throat ≥3 days earlier than in the nose, and up to 7 days earlier for 1 individual [10].

In a separate study of individuals who underwent intranasal inoculation with SARS-CoV-2, 10 of 18 participants (55%) with sustained infection had detectable virus in the throat for ≥1 day before virus was detectable in the nose with polymerase chain reaction (PCR) testing [11]. Notably, replication-competent (infectious) virus was successfully cultured from throat swab specimens before nasal swab specimens in 12 of these 18 individuals (67%). These data suggest that if only nasal swab specimens are used, even molecular COVID-19 tests with high analytical sensitivity (including tests with low limits of detection; down to 1000 copies/mL) could yield false-negative results for individuals who may be capable of transmitting SARS-CoV-2 [12].

Analyses of paired viral load dynamics from the cohort with naturally acquired infection suggested that using combined throat-nasal swab specimens rather than nasal swab specimens alone with a high-analytical-sensitivity molecular COVID-19 tests would improve clinical sensitivity by >40% during the first days of SARS-CoV-2 infection [10]. However, because a subset of individuals may present with rising viral loads in the nose before the throat, combination throat-nasal swab tests are likely to yield higher clinical sensitivity than throat swab specimens alone. Indeed, the current Infectious Diseases Society of America Guidelines on the diagnosis of COVID-19 [13] recommend against the use of throat swab specimens alone for both molecular diagnostic tests [14] and Ag-RDTs [15].

Cross-sectional analyses of participant populations later in infection (after the first few days) are unlikely to observe the benefit of combining throat-nasal swabbing on Ag-RDT clinical sensitivity. For example, reanalyzing viral loads cross-sectionally between days 0 and 12 of infection in our group’s study [5] predicted only a marginal benefit (6%) for combined throat-nasal swab Ag-RDT over the observed clinical sensitivity of nasal-specimen-only Ag-RDT (43%). This small, predicted benefit is similar to that observed in a later cross-sectional study of 96 infected individuals in San Francisco [9]. In that study, combined throat-nasal Ag-RDT increased clinical sensitivity from 54% (for nasal-specimen-only Ag-RDT) to 59% [9]. We note that the high PCR positivity rate (83%) among the 115 participants screened may suggest a study population skewed toward later infection. The clinical sensitivity of combined throat-nasal Ag-RDT may also be influenced by the throat swab specimen collection technique [16] or by whether a test designed for use with nasal swab specimens exhibits lower analytical sensitivity when used with throat swab specimens [13, 17].

Maximizing the clinical sensitivity of COVID-19 tests—both Ag-RDTs and molecular diagnostic tests—for early detection is paramount, particularly given surges in emerging variants with potential for evasion of humoral immunity [18]. To improve performance, Ag-RDTs and molecular COVID-19 tests need to be analytically and clinically validated by manufacturers for use with combination throat-nasal swab specimens, including clinical validation studies on (at least) symptomatic patient specimens. This combination throat-nasal swab test could use a single swab sampling both the throat and the nose or (to address consumer hesitancy) separately collected swab specimens from the nose and throat, which could be placed into the same elution medium.

Based on past FDA flexibilities offered for the validation of COVID-19 tests for emergency use authorizations (Supplementary Table), the FDA is likely to accept noninferiority studies, perhaps even only in symptomatic patients (historically, approximately 30 positive and 30 negative results are required for the emergency use authorization). For clearance, the FDA may accept evaluation of the combined throat-nasal swab specimen against a standard single swab specimen, showing at least in symptomatic patients that the combination specimen is not inferior (has equivalent or better sensitivity) in the requisite number of patients with positive results, usually 120 with positive and 500 with negative results for an over-the-counter test. The best way for developers to determine what the FDA expects is through the Q-Submission process [19], which is a no-charge FDA submission. The developers can ask their questions of the FDA and receive a response within 70 calendar days [19].

Although it may not be required for test validations, it would be particularly useful for studies to include populations for whom early detection has the most impact, such as the immunocompromised and those residing in congregate settings (eg, skilled nursing facilities and dormitories). These populations would demonstrate just how useful combination throat-nasal swab specimens are for populations at high risk of transmission or severe disease. We also suggest studies to investigate whether the use of combined throat-nasal swab specimens provide similar benefit for diagnostic testing of other upper respiratory viral infections, such as influenza and respiratory syncytial virus.

## Supplementary Data

Supplementary materials are available at *Clinical Infectious Diseases* online. Consisting of data provided by the authors to benefit the reader, the posted materials are not copyedited and are the sole responsibility of the authors, so questions or comments should be addressed to the corresponding author.

## Supplementary Material

ciae381_Supplementary_Data

## Notes

***Financial support*.** This work was supported by the Bill & Melinda Gates Foundation (grant INV-023124) and the Ronald and Maxine Linde Center for New Initiatives at the California Institute of Technology. A. V. W. is supported by the National Institutes of Health (NIH) F30AI167524 and NIH T32GM008042 to the UCLA-Caltech Medical Scientist Training Program.
