# Supplementary material for: Validating Combination Throat-Nasal Swab Specimens for COVID-19 Tests Would Improve Early Detection, Especially for the Most Vulnerable
Source: Clin Infect Dis. 2024 Jul 23;79(4):815–8. doi: 10.1093/cid/ciae381 (PMC11478585; doi:10.1093/cid/ciae381)
Supplement: ciae381_Supplementary_Data [file ciae381_supplementary_data.docx]

**Supplementary Appendix for:**

**Validating combination throat-nasal swabs for COVID-19 tests would improve early detection, especially for the most vulnerable**

Alexander Viloria Winnett, BSc.^1,2^, Timothy Stenzel, MD, PhD^3^, Rustem F. Ismagilov, PhD^1*^

1. California Institute of Technology, Division of Chemistry and Chemical Engineering; Division of Biology & Biological Engineering
2. University of California Los Angeles – California Institute of Technology Medical Scientist Training Program
3. Former Director, Office of In Vitro Diagnostics, United States Food and Drug Administration

* Corresponding author: [rustem.admin@caltech.edu](mailto:rustem.admin@caltech.edu); 626-395-8130

Alternate Corresponding author: [awinnett@caltech.edu](mailto:awinnett@caltech.edu); 781-985-1502

**Table of Contents**

Supplemental Table

**Supplemental Table.** Demonstration of flexibilities exhibited by the U.S. Food and Drug Administration (FDA) in the emergency use authorization (EUA) of COVID-19 tests.

|  | **Title** | **Description of flexibility** | **Reference** |
| --- | --- | --- | --- |
| 1 | Authority for Emergency Use Authorization (EUA) | Under law, the EUA authorities allow a lower bar for test development and validation. The FDA made full use of this flexibility during the COVID-19 pandemic, as well as in other emergencies. | <https://www.fda.gov/regulatory-information/search-fda-guidance-documents/emergency-use-authorization-medical-products-and-related-authorities> |
| 2 | Validation specimen type | With few patient specimens available early in the COVID-19 pandemic, contrived positive specimens were used for clinical evaluation. | <https://www.fda.gov/media/136112/download?attachment>  (See *Clinical evaluation*) |
| 3 | Scaling of manufacturing | To accommodate emergency response needs in the production of COVID-19 EUA tests, the FDA is permitted to waive otherwise-applicable current good manufacturing practice (CGMP) requirements (e.g., storage or handling). | <https://www.fda.gov/regulatory-information/search-fda-guidance-documents/emergency-use-authorization-medical-products-and-related-authorities>  (See Section IV.C) |
| 4 | Specimen Pooling | To increase capacity, pooling of specimens for testing by a previously authorized test was allowed without FDA review of pooled specimen performance, if single specimen validation data supported compatibility. | <https://www.fda.gov/medical-devices/covid-19-emergency-use-authorizations-medical-devices/in-vitro-diagnostics-euas-molecular-diagnostic-tests-sars-cov-2#amendment>  (See *Pooling and Serial Testing Amendment for Certain Molecular Diagnostic Tests for SARS-CoV-2*) |
| 5 | Extension of test expiration dates | Expiration dates for at-home, over-the-counter (OTC) COVID-19 tests were extended when test manufacturers provided data demonstrating a longer shelf-life than was known when the test was first authorized. | <https://www.fda.gov/medical-devices/coronavirus-covid-19-and-medical-devices/home-otc-covid-19-diagnostic-tests>  (See *Authorized At-Home OTC COVID-19 Diagnostic Tests and Expiration Dates*)  <https://www.fda.gov/regulatory-information/search-fda-guidance-documents/emergency-use-authorization-medical-products-and-related-authorities#expdate>  (See Section IV.B) |
| 6 | Multi-analyte tests | Multi-analyte (multi-pathogen) tests were authorized under COVID-19 EUA. | <https://www.fda.gov/media/176728/download?attachment> |
| 7 | Performance accounting for study population | Positive Percent Agreement during clinical evaluation was modeled to adjust for the viral load of participants in the study population. | <https://www.fda.gov/media/157544/download?attachment>  (See *Section 2.6*) |
| 8 | At-home testing | Simulated home test environments for over-the-counter (OTC) test validation were considered. | <https://www.fda.gov/media/157544/download?attachment>  (See *Section 2.6*) |
| 9 | Asymptomatic screening | Screening of asymptomatic patients using tests were allowable for tests that did not initially include asymptomatic claims. | <https://www.fda.gov/medical-devices/covid-19-emergency-use-authorizations-medical-devices/in-vitro-diagnostics-euas-antigen-diagnostic-tests-sars-cov-2#SerialTesting>  (See *Antigen EUA Revisions for Serial (Repeat) Testing*) |
| 10 | Serial testing | The FDA exhibited flexibility to allow asymptomatic claims with serial testing. | <https://www.fda.gov/medical-devices/covid-19-emergency-use-authorizations-medical-devices/in-vitro-diagnostics-euas>  (See *Umbrella EUA for SARS-CoV-2 Molecular Diagnostic Tests for Serial Testing* and *Antigen EUA Revisions for Serial (Repeat) Testing*) |
| 11 | Performance with serial testing | Cumulative Positive Percent Agreement through serial testing, rather than one-time testing, was considered in the review of test performance for EUA. | <https://thehill.com/opinion/healthcare/515628-fda-were-constantly-working-on-covid-testing-options/>  <https://www.fda.gov/medical-devices/covid-19-emergency-use-authorizations-medical-devices/in-vitro-diagnostics-euas-antigen-diagnostic-tests-sars-cov-2#SerialTesting> |
